# Efficacy and Adverse Events of PD-1 Inhibitors in Patients With Advanced Urothelial Carcinoma From a Real-World Experience

**DOI:** 10.3389/fphar.2022.837499

**Published:** 2022-03-18

**Authors:** Fengze Sun, Dawei Wang, Aina Liu, Tianqi Wang, Dongxu Zhang, Huibao Yao, Kai Sun, Zhongbao Zhou, Guoliang Lu, Jitao Wu

**Affiliations:** ^1^ Department of Urology, Yantai Yuhuangding Hospital, Qingdao University, Yantai, China; ^2^ Department of Urology, Ruijin Hospital, Shanghai Jiaotong University School of Medicine, Shanghai, China; ^3^ Department of Oncology, Yantai Yuhuangding Hospital, Qingdao University, Yantai, China; ^4^ Department of Urology, Beijing Tiantan Hospital, Capital Medical University, Beijing, China

**Keywords:** urothelial carcinoma, programmed death -1/programmed death ligand 1 (PD-1/PD-L1), adverse events, chemotherapy, efficacy

## Abstract

**Background:** Programmed death 1 (PD-1) inhibitors—tislelizumab, toripalimab, camrelizumab, and sintilimab—are used for advanced urothelial carcinoma (UC) in China. To date, the efficacy and adverse events (AEs) of these PD-1 inhibitors have been poorly reported for advanced UC.

**Methods:** We reviewed 118 patients treated with PD-1 inhibitors for advanced UC from July 2019 to October 2021 at Yantai Yuhuangding Hospital. Patient data were obtained from hospital records and telephone follow-ups. The safety and efficacy of PD-1 inhibitors were assessed by RESIST and Common Terminology Criteria for Adverse Events (version 4.0), respectively.

**Results:** During a median follow-up period of 6 months, 112 patients (95%) experienced AEs; of these, 104 (88%) were grade 1–2 AEs, and 60 (51%) were grade 3–4 AEs. The most common AE was anemia, and no patients died as a result of treatment. A subanalysis according to treatment method (PD-1 inhibitor vs. PD-1 inhibitor plus chemotherapy) was performed. The incidence of grade 1–2 AEs was not different between the groups (85% vs. 94%), but combination therapy significantly increased grade 3–4 AEs (32% vs. 89%). Monotherapy and combination therapy also did not differ with regard to immune-related AEs of grades 1–2 (13% vs. 22%) or grades 3–4 (1% vs. 6%). In efficacy, complete response was not observed, but 33 patients (28%) had partial response, 30 (25%) had stable disease, and 47 had progressive disease (40%). The overall response and disease control rates were 28% and 53%, respectively. The preliminary efficacy of disease control was better with combination therapy versus monotherapy (78 vs. 43%).

**Conclusion:** PD-1 inhibitors show promising tolerance and efficacy in advanced UC. PD-1 inhibitors combined with chemotherapy offered better disease control but had more grade 3–4 AEs. The clinical use of combination therapy warrants caution.

## Introduction

The prognosis of urothelial carcinoma (UC) worsens as tumors progress, with a 5-year survival rate of less than 5% for distant metastases (Apetoh et al., 2015). The first-line treatment of advanced UC has been platinum-based, especially cisplatin-based chemotherapy (von der Maase et al., 2005; De Santis et al., 2012). For cisplatin-ineligible patients, carboplatin-based chemotherapy is an option. The efficacy of platinum-based treatment has not improved substantially in recent years.

Immune checkpoint inhibitors (ICIs) have become important as treatment for advanced UC (Johnson et al., 2017); they include programmed death ligand 1 (PD-L1) and programmed death 1 (PD-1) inhibitors. Many clinical trials have shown that ICI monotherapy has better efficacy and tolerability than chemotherapy (Massard et al., 2016; Balar et al., 2017a; Apolo et al., 2017; Balar et al., 2017b; Bellmunt et al., 2017; Sharma et al., 2017; Powles et al., 2018). The latest research shows that combination treatment with an ICI and chemotherapy improves progression-free survival (PFS) and overall survival (OS) more than monotherapy (Galsky et al., 2020; Chen et al., 2021; Powles et al., 2021). These findings have provided a new treatment method in clinical practice.

Despite the impressive efficacy of ICIs, treatment-related adverse events (AEs) should not be neglected. During immune regulation by ICIs, some normal tissue can be wrongly attacked, causing immune-related adverse events (irAEs) (Wang et al., 2018). Organ-specific irAEs, including colitis, hepatitis, pneumonitis, and hypothyroidism, as well as general AEs related to immune activation, including fatigue, diarrhea, and rash, have been common and may negatively impact quality of life. In general, the toxicity of ICIs is less than that of chemotherapy (Abdel-Rahman et al., 2015; Abdel-Rahman and Fouad, 2016a; Abdel-Rahman and Fouad, 2016b).

At present, four PD-1 inhibitors—tislelizumab, toripalimab, camrelizumab, and sintilimab—have been applied in National Medicare with lower price. Tislelizumab and toripalimab have resulted in encouraging outcomes for advanced UC in clinical trials (Ye et al., 2021; Sheng et al., 2022), and they were approved by the National Medical Products Administration for advanced UC. Camrelizumab and sintilimab have also shown beneficial effects in some cases (Cao et al., 2021; Ni et al., 2021; Wang et al., 2021).

As ICI use has increased in patients with cancer, management of AEs has become an indispensable part of clinical practice. Although these PD-1 inhibitors have played an important role in the treatment of advanced UC, reporting of related AEs in advanced UC remained rare. In this real-world retrospective clinical study, we analyzed the AEs of ICIs in advanced UC to improve their recognition and management.

## Methods

### Patient Selection and Procedures

We retrospectively enrolled patients who had advanced or metastatic UC as confirmed by histology and radiography. Patients had at least one measurable lesion used for RECIST classification, and an Eastern Cooperative Oncology Group (ECOG) performance score of 0–1 was required. Patients received one of the following anti-PD-1 agents: tislelizumab, toripalimab, camrelizumab, or sintilimab. Some patients received PD-1 inhibitor monotherapy; other patients received a PD-1 inhibitor and a platinum-based chemotherapy, for which cisplatin, carboplatin, or other platinum was selected according to the patient’s status.

All clinical data were obtained by telephone follow-up and from electronic medical records. The research was approved by the Ethics Committee of Yantai Yuhuangding Hospital, and individual consent from patients for this retrospective analysis was waived.

### Outcomes

Tolerability was our analysis points for evaluation using the National Cancer Institute Common Terminology Criteria for Adverse Events, version 4.0. The AEs were classified by two individual reviewers. The efficacy of treatment was also recorded. Measurable disease was documented before treatment was initiated, and the outcomes were confirmed by comparing initial and follow-up imaging. Tumor response was evaluated using RECIST 1.1. Objective tumor responses included complete response (CR: the disappearance of all target lesions), partial response (PR: at least a 30% reduction in the sum of the diameters of the target lesions), stable disease (SD: the lesions change between PR and progressive disease [PD]), and PD (a 20% increase in the sum of the diameters of the target lesions or appearance of new lesions). The CR and PR were defined together as the objective response rate (ORR); the CR, PR, and SD were defined together as the disease control rate (DCR).

### Statistical Analyses

Descriptive statistics (percentages, means, and medians) were used to report baseline characteristics and AEs of patients. Categorical variables were analyzed using a chi-squared test or Fisher’s exact test. p values of <0.05 were considered significant. Statistical analyses were performed using SPSS statistical software, version 20.0 (IBM, Armonk, NY, United States).

## Results

### Basic Characteristics of Patients

Overall, 118 patients were enrolled in this real-world study from July 2019 to October 2021. Patient characteristics are presented in Table 1. The median age was 68 years (range, 51–85 years), and most patients were men (68%). ECOG performance scores were mostly 1 (53%). 62 patients (53%) were diagnosed with UC in the bladder; 34 (29%), in the renal pelvis; and 22 (19%), in the ureter. 77 patients (65%) had visceral metastasis, 29 (25%) had liver metastasis, and 20 (17%) had only lymph node metastasis. Before PD-1 inhibitor treatment, 53 patients (45%) did not receive any treatment; 56 patients (47%) received one platinum-based therapy, and 9 patients (8%) received at least two platinum-based therapies. PD-L1 expression was tested in 28 patients, of which 14 patients were positive and 14 were negative. 70 patients (59%) received tislelizumab, 25 (21%) received camrelizumab, 18 (15%) received toripalimab, and only 5 (4%) received sintilimab. 82 patients (69%) received a PD-1 inhibitor alone, and 36 patients received the combination of a PD-1 inhibitor and chemotherapy.

**TABLE 1:** The patients’ baseline disease characteristics.

Characteristics	All patients (*n* = 118)
Age, Y	
Median (range)	68 (51–85)
Gender, *n* (%)	
Male	80 (68%)
Female	38 (32%)
Smoking status, n (%)	
Never	52 (44%)
Current	21 (18%)
Former	45 (38%)
ECOG performance at baseline, n (%)	
0	55 (47%)
1	63 (53%)
Site of primary tumor, n (%)	
Bladder	62 (53%)
Renal pelvis	34 (29%)
Ureter	22 (19%)
Known metastasis at baseline, n (%)	
Visceral metastasis	77 (65%)
Liver metastasis	29 (25%)
Only lymph node	20 (17%)
Number of prior regimens of anticancer therapies, n (%)	
0	53 (45%)
1	56 (47%)
≥2	9 (8%)
PD-L1 expression, n (%)	
Positive	14 (12%)
Negative	14 (12%)
Unknown	90 (76%)
Anti-PD-1 mAbs, n (%)	
Tislelizumab	70 (59%)
Camrelizumab	25 (21%)
Toripalimab	18 (15%)
Sintilimab	5 (4%)
Treatment method	
PD-1 inhibitor	82 (69%)
PD-1 inhibitor and chemotherapy	36 (31%)

### Adverse Event

In our study, 112 patients (95%) experienced AEs; of these, 104 patients (88%) experienced grade 1–2 AEs (Table 2). The most common AEs were anemia (32%) and decreased appetite (19%). Sixty patients (51%) experienced grade 3–4 AEs, of which anemia (10%), decreased neutrophil count (9%) and decreased white blood cell count (6%), and urinary tract infection (5%) occurred in at least 5% of the population. Seven patients (6%) discontinued PD-1 inhibitors because of AEs, and no patient died as a result of treatment. In our study, 19 patients (16%) experienced grade 1–2 irAEs; these included mostly skin reactions (7%), hypothyroidism (5%), and hyperthyroidism (3%) (Table 3). Only three patients (3%) experienced grade 3–4 AEs, of which two patients (2%) had skin reactions and one patient (1%) had colitis. Moreover, the median time of irAE was 9 weeks.

**TABLE 2:** Treatment-related adverse events occurring in patients.

	All patients (*n* = 118)	
	Grade 1–2	Grade 3–4
Any adverse events	104 (88%)	60 (51%)
Adverse events leading to discontinuation	7 (6%)	
Anemia	38 (32%)	12 (10%)
Decreased appetite	22 (19%)	5 (4%)
Increased ALT	21 (18%)	4 (3%)
Pyrexia	21 (18%)	3 (3%)
Increased AST	20 (17%)	3 (3%)
Pruritus	18 (15%)	1 (1%)
Rush	18 (15%)	2 (2%)
Decreased neutrophil count	18 (15%)	11 (9%)
Constipation	17 (14%)	3 (4%)
Fatigue	17 (14%)	5 (4%)
Decreased white blood cell count	16 (14%)	7 (6%)
Nausea	16 (14%)	2 (2%)
Urinary tract infection	14 (12%)	6 (5%)
Proteinuria	11 (9%)	1 (1%)
Thrombocytopenia	11 (9%)	4 (3%)
Vomiting	11 (9%)	2 (2%)
Reactive capillary hemangiomas	10 (8%)	1 (1%)
Hypothyroidism	10 (8%)	0
Diarrhea	10 (8%)	2 (2%)
Increased blood bilirubin	10 (8%)	1 (1%)
Increased blood urea	9 (8%)	2 (2%)
Increased blood alkaline phosphatase	9 (8%)	3 (4%)
Increased gamma-glutamyl transferase	9 (8%)	2 (2%)
Asthenia	9 (8%)	1 (1%)
Hyponatremia	8 (7%)	3 (4%)
Hypoalbuminemia	8 (7%)	1 (1%)
Upper respiratory tract infection	8 (7%)	3 (4%)
Arthralgia	7 (6%)	1 (1%)
Hematuria	7 (6%)	3 (4%)
Hyperthyroidism	4 (3%)	0

**TABLE 3:** Immune-treatment adverse events occurring in patients.

	All patients (*n* = 118)	
	Grade 1–2	Grade 3–4
Any immune-related adverse events	19 (16%)	3 (3%)
Skin adverse reaction	8 (7%)	2 (2%)
Hypothyroidism	6 (5%)	0
Hyperthyroidism	3 (3%)	0
Colitis	1 (1%)	1 (1%)
Pneumonitis	1 (1%)	0
Hepatitis	1 (1%)	0
Myocarditis	1 (1%)	0
Myasthenia gravis	1 (1%)	0
Time to happen (week)		
Median (range)	9 (1–31)	

**FIGURE 1:**
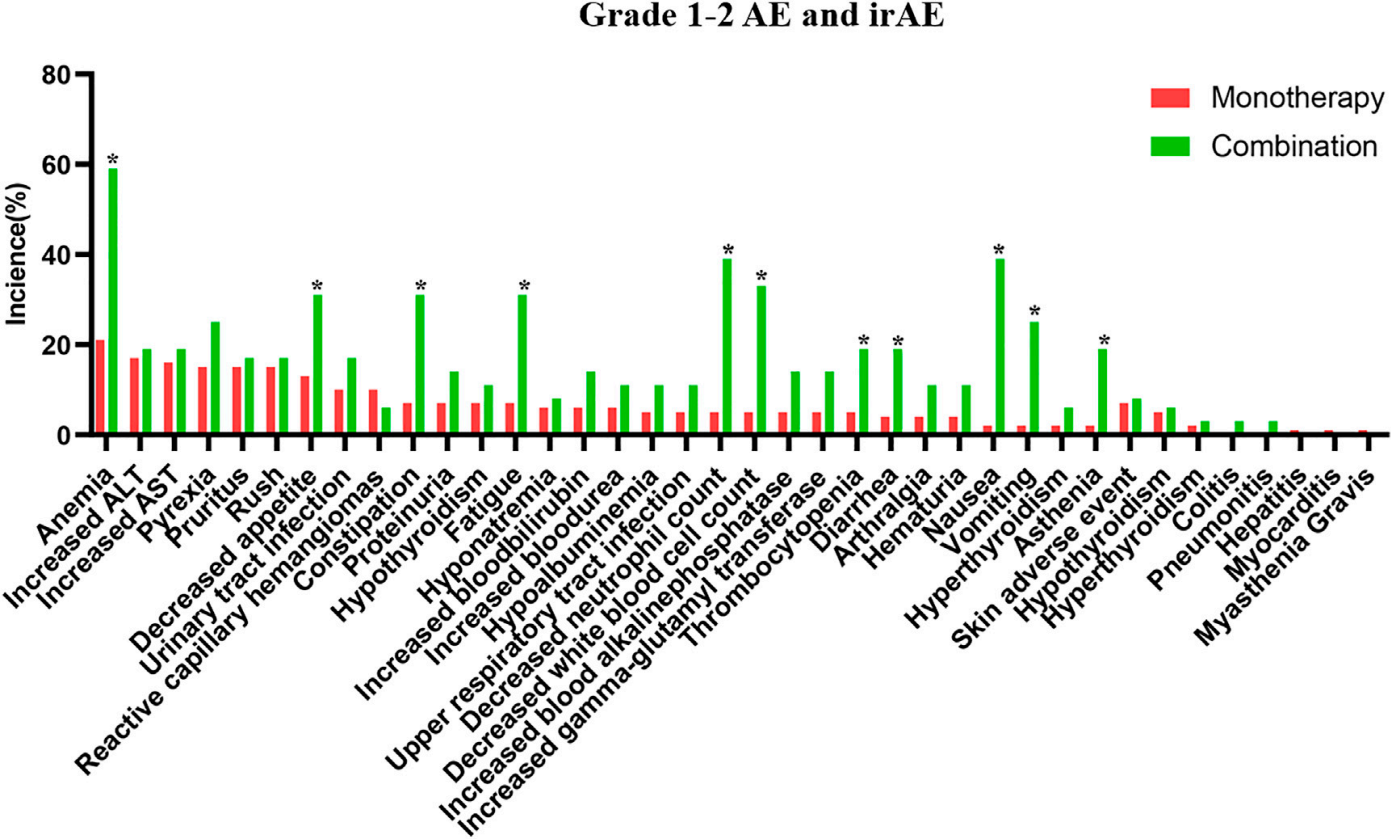
The detailed grade 1–2 adverse events in PD-1 inhibitor and PD-1 inhibitor plus chemotherapy.

**FIGURE 2:**
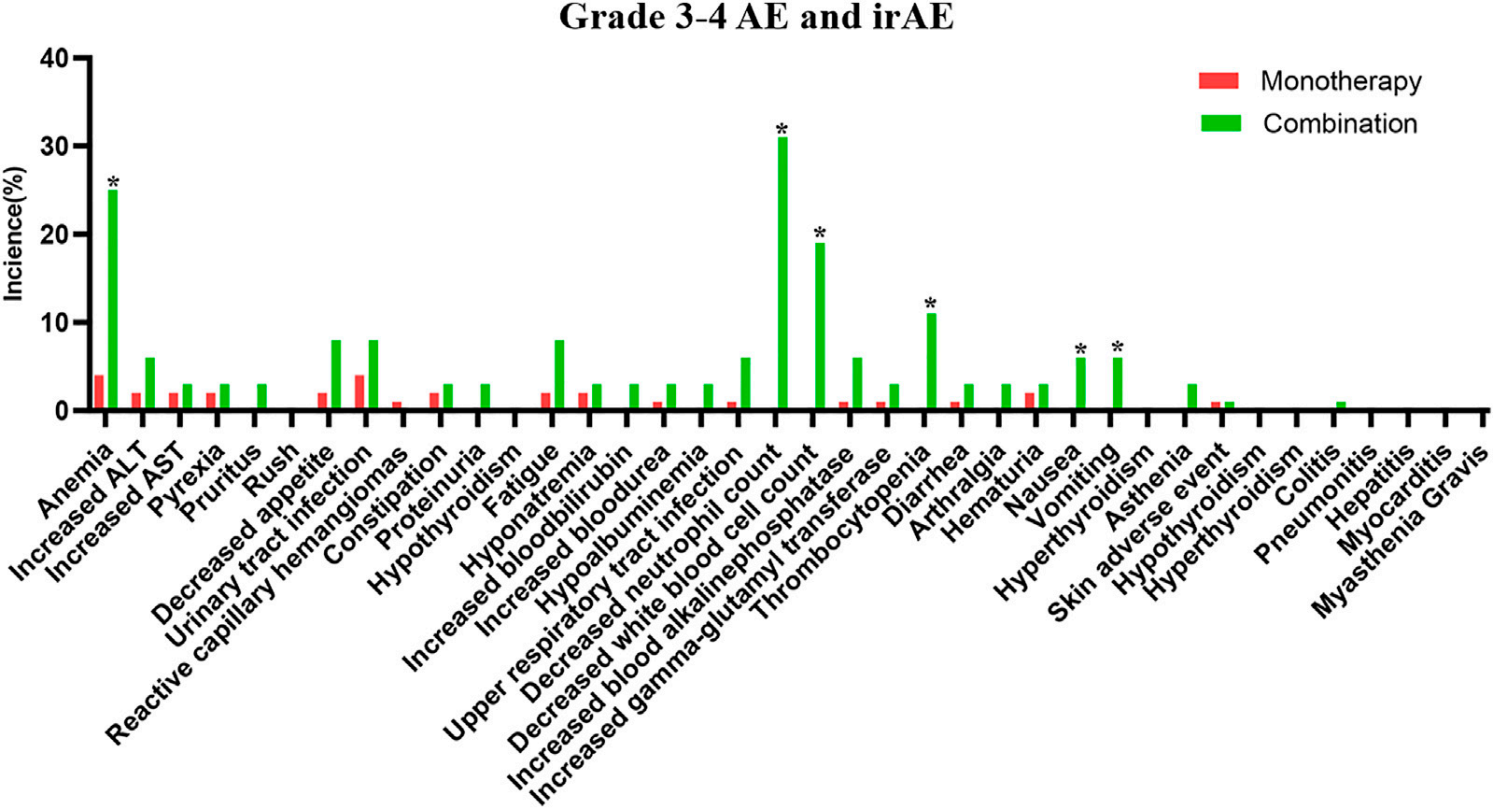
The detailed grade 3–4 adverse events in PD-1 inhibitor and PD-1 inhibitor plus chemotherapy.

The safety of the PD-1 inhibitors was also analyzed according to treatment method (in combination or monotherapy). The detailed characters of two groups are shown in Supplementary Table S1. Patients experienced mostly grade 1–2 AEs and irAEs with monotherapy or with combination therapy, and no difference was noted between the two groups (*p* > 0.05). Details about AEs are shown in Figure 1 and Supplementary Table S2. Eleven AEs, including anemia, decreased appetite, and constipation, occurred more frequently with combination therapy than with monotherapy. The most frequent grade 1–2 irAEs in both groups were skin reactions and hypothyroidism. Thirty-two patients (89%) experienced grade 3–4 AEs with combination therapy, but fewer patients (32%) experienced these with monotherapy (*p* < 0.01). The combination therapy mainly increased the myelosuppressive response, as seen by the decreased neutrophil count, anemia, decreased white blood cell count, and thrombocytopenia (Figure 2). Only one patient experienced a grade 3–4 skin reaction with monotherapy; two patients experienced grade 3–4 skin reactions and colitis with combination therapy. Moreover, the median time of irAE in monotherapy and combination therapy was 8.5 and 9 weeks, respectively (*p* > 0.05). Three patients (4%) discontinued monotherapy because of AEs, whereas four patients (11%) could not tolerate serious AEs and discontinued combination therapy (*p* > 0.05).

### Efficacy

During the treatment period, the best response of each patient was recorded. As shown in Table 4, 33 patients (28%) experienced a PR, 30 (25%) experienced SD, 47 (40%) developed PD, and none experienced a CR, yielding an ORR of 28% and a DCR of 53%. In the subanalysis, the ORR was 24% and the DCR was 43% for monotherapy; for combination therapy, the ORR was 36% and the DCR reached 78%. The combination therapy showed a better DCR compared with monotherapy (*p* < 0.05).

**TABLE 4:** Disease response in PD-1 inhibitor and combination.

Efficacy	Monotherapy (*n* = 82)	Combination (*n* = 36)	All patients (*n* = 118)
Complete response, n (%)	0	0	0
Partial response, n (%)	20 (24%)	13 (36%)	33 (28%)
Stable disease, n (%)	15 (18%)	15 (42%)	30 (25%)
Progressive disease, n (%)	40 (49%)	7 (19%)	47 (40%)
Not evaluable for response, n (%)	7 (9%)	1 (3%)	8 (7%)
ORR (%, CR + PR)	24%	36%	28%
DCR (%, CR + PR + SD)	43%	78%	53%

## Discussion

In advance or metastatic UC, cis-platinum-based chemotherapy is still considered the first-line treatment. With the rapid development of ICIs, many clinical trials have identified more important roles for these drugs in the treatment of UC. At present, four PD-1 inhibitors have been approved for clinical use: tislelizumab, toripalimab, camrelizumab, and sintilimab. Although only tislelizumab and toripalimab are approved for advanced UC, more drugs are being explored clinically, and their AEs may become obstacles for clinical application. In advanced UC, the tolerance and efficacy of the four existing PD-1 inhibitors have been reported only rarely.

In this real-world retrospective study, we found—to our knowledge for the first time—that the four available PD-1 inhibitors were safe and had acceptable tolerability in patients with advanced UC. Most patients experienced grade 1–2 AEs. Anemia was the most common, and it was caused mainly by tislelizumab, as in previous studies (Cao et al., 2021). Reactive capillary hemangioma is the most specific AE of camrelizumab, as in other published study (Chen et al., 2019). Sixty patients in our study experienced grade 3–4 AEs which was more than previous studies (Ye et al., 2021). Additional chemotherapy could significantly increase grade 3–4 AEs (Li et al., 2020). Therefore, we performed a subanalysis according to the treatment method (PD-1 inhibitor vs. PD-1 inhibitor plus chemotherapy) to identify differences.

The irAEs are regarded as specific to ICIs. In the tumor microenvironment, the cancer cell surfaces have PD-L1 receptors that interact with immune cells to suppress the immune system. The function of PD-1 inhibitors is to competitively bind PD-1, keep the immune cells activated, and achieve antitumor functions. When PD-1 is blocked, T cells shift toward pro-inflammatory Th1 and Th17 cells, contributing to autoimmunity (Luger et al., 2008; Dulos et al., 2012). In our study, 19 patients experienced grade 1–2 irAEs, which were mainly skin reactions and hypothyroidism. Only three patients experienced grade 3–4 AEs, which were skin reactions and colitis. The toxicities with high fatality rates, such as myocarditis and pneumonitis, are usually difficult to detect (Wang et al., 2018). In our study, one patient, who had a history of coronary heart disease, experienced mild myocarditis, and one patient experienced mild pneumonitis accompanied by lung metastasis; these experiences suggested that the irAEs were associated with basic disease and with metastasis, as reported in previous studies (Drobni et al., 2020; Pozzessere et al., 2020; Pirozzi et al., 2021). It should be paid attention that the treatment of basic disease could be related with the occurrence of related irAEs in the period of ICI treatment.

In the subanalysis, we found that AEs experienced with either monotherapy or combination therapy were mostly grades 1–2. The most common AEs associated with monotherapy were anemia, transaminase elevation. Moreover, the incidence of grade 1–2 adverse reactions was 85%, which was similar to 94% in NCT04004221 and 85% in POLARIS-03 (Ye et al., 2021; Sheng et al., 2022) and more than 46% in CheckMate 275 and 63.9% in MEDI4736 (Bellmunt et al., 2017; Sharma et al., 2017). The domestic PD-1 inhibitors had a higher incidence of AEs than abroad PD-1 inhibitors, as in another published study (Li et al., 2020). In combination therapy, the most common adverse effects were myelosuppressive responses, including neutropenia, anemia, leukopenia, and thrombocytopenia. The occurrence reached 94%, which was similar to KEYNOTE-361 (Powles et al., 2021). No significant difference was observed between rates of grade 1–2 AEs with monotherapy and combination therapy. Grade 3–4 AEs occurred more often with combination therapy than with monotherapy, as seen in previous studies (Galsky et al., 2020; Powles et al., 2021). Anemia and urinary tract infection were common with monotherapy, but the myelosuppressive response was observed mostly with combination therapy. The most common grade 3–4 AEs could be immune related and include fatal diseases like pneumonitis, dyspnea, diarrhea, colitis, ALT or AST increase, and hepatitis (Wang et al., 2019). Clinical vigilance is necessary for early recognition and intervention to prevent severe AEs.

With regard to irAEs, combination therapy and monotherapy did not show obvious differences. Skin reactions and hypothyroidism were common, which was similar to some previous reports (Baxi et al., 2018; Sibaud, 2018). The incidence of irAE was low in our study, potentially because of the short follow-up time. Hepatitis can occur after 34 weeks of exposure to nivolumab (Imafuku et al., 2017), and the median time to onset of late irAEs was 16.6 months in a multicenter study (Nigro et al., 2020). Therefore, assessment of irAEs requires longer follow-up times.

In the efficacy analysis, we found that combination therapy had a better DCR than monotherapy, whereas the ORR was not different. High PD-L1 expression was related to better efficacy, as previously reported (Lin et al., 2018; Shen and Zhao, 2018), and PD-L1 expression without selection could limit efficacy. In addition, OS and PFS were not determined, because the follow-up treatment schemes were not the same for patients within the groups, especially in the combination therapy arm. We could not conclude that the efficacy was better in combination therapy.

There are also many limitations to this study. The most important one is that our analysis was limited by retrospective data. Although we attempted to identify all relevant AE information through medical records and telephone follow-ups, some information might be forgotten and neglected by patients or their family members, so the incidence of AEs could be underestimated. With limitation of patients in toripalimab, camrelizumab, and sintilimab, the AEs in different PD-1 inhibitors were not compared. Because we focused on AEs, the follow-up treatment of included patients was not screened, and OS and PFS were not assessed. Thus, the long-term effectiveness of combination therapy and monotherapy cannot be fully analyzed. Fewer patients were treated with a PD-1 inhibitor plus chemotherapy, which caused bias in the analysis of the results. The findings of this study must be verified by larger clinical trials.

## Conclusion

In this real-world retrospective study, we described the safety and efficacy of four PD-1 inhibitors used to treat advanced UC in our institution. Generally, ICI-related toxicities were mild, safe, and tolerable. In a subanalysis of ICI monotherapy versus combination therapy with chemotherapy, the combination of a PD-1 inhibitor and chemotherapy showed more grade 3–4 AEs than monotherapy. However, the numbers of patients in the two groups who discontinued treatment because of AEs were not significantly different, and no deaths related to AEs were observed. The DCR was better with combination therapy than with monotherapy. However, because of the high rate of grade 3–4 AEs, combination therapy should be used cautiously in clinical practice. The conclusions from this study should be confirmed by large-scale clinical trials.

## Data Availability

The raw data supporting the conclusion of this article will be made available by the authors, without undue reservation.
