# Supplementary material for: Efficacy and Adverse Events of PD-1 Inhibitors in Patients With Advanced Urothelial Carcinoma From a Real-World Experience
Source: Front Pharmacol. 2022 Mar 18;13:837499. doi: 10.3389/fphar.2022.837499 (PMC8971813; doi:10.3389/fphar.2022.837499)
Supplement: Supplementary file 1 [file Table1.docx]

| Characteristics | Monotherapy(n=82) | Combination(n=36) |
| --- | --- | --- |
| Age, Y |  |  |
| Median (range) | 68 (51-85) | 67(57-76) |
| Gender, n (%) |  |  |
| Male | 55(67%) | 25(69%) |
| Female | 27(33%) | 11(31%) |
| Smoking status, n (%) |  |  |
| Never | 35(43%) | 17(47%) |
| Current | 14(17%) | 7(19%) |
| Former | 33(40%) | 12(33%) |
| ECOG performance at baseline, n (%) |  |  |
| 0 | 35(43%) | 20(56%) |
| 1 | 47(57%) | 16(44%) |
| Site of primary tumor, n (%) |  |  |
| Bladder | 46(56%) | 16(44%) |
| Renal pelvis | 20(24%) | 14(39%) |
| Ureter | 16(20%) | 6(17%) |
| Known metastasis at baseline, n (%) |  |  |
| Visceral metastasis | 51(62%) | 26(72%) |
| Liver metastasis | 24(29%) | 5(14%) |
| Only lymph node | 12(15%) | 8(22%) |
| Number of prior regimens of anticancer therapies, n (%) |  |  |
| 0 | 34(41%) | 19(52%) |
| 1 | 40(48%) | 16(44%) |
| ≥2 | 8(10%) | 1(3%) |
| PD-L1 expression, n (%) |  |  |
| Positive | 10(12%) | 4(11%) |
| Negative | 11(13%) | 3(8%) |
| Unknown | 61(74%) | 29(81%) |
| Anti-PD-1 mAbs, n (%) |  |  |
| Tislelizumab | 50(61%) | 20(56%) |
| Camrelizumab | 17(21%) | 8(22%) |
| Toripalimab | 12(15%) | 6(17%) |
| Sintilimab | 3(4%) | 2(6%) |
| Anti-PD-1 mAbs plus chemotherapy, n (%) |  |  |
| Cisplatin-based |  | 17(47%) |
| Carboplatin-based |  | 9(25%) |
| Other platinum-based |  | 10(28%) |

**Supplementary Table S1** The detailed patients baseline disease characteristics in monotherapy and combination therapy.
