# Supplementary material for: Efficacy and Adverse Events of PD-1 Inhibitors in Patients With Advanced Urothelial Carcinoma From a Real-World Experience
Source: Front Pharmacol. 2022 Mar 18;13:837499. doi: 10.3389/fphar.2022.837499 (PMC8971813; doi:10.3389/fphar.2022.837499)
Supplement: Supplementary file 2 [file Table2.docx]

|  | Monotherapy(n=82) | | Combination(n=36) | |
| --- | --- | --- | --- | --- |
|  | Grade 1-2 | Grade 3-4 | Grade 1-2 | Grade 3-4 |
| Any adverse events | 70(85%) | 28(32%) | 34(94%) | 32(89%) |
| Adverse events leading to discontinuation | 3(4%) | | 4(11%) | |
| Anemia | 17(21%) | 3(4%) | 21(59%) | 9(25%) |
| Increased ALT | 14(17%) | 2(2%) | 7(19%) | 2(6%) |
| Increased AST | 13(16%) | 2(2%) | 7(19%) | 1(3%) |
| Pyrexia | 12(15%) | 2(2%) | 9(25%) | 1(3%) |
| Pruritus | 12(15%) | 0 | 6(17%) | 1(3%) |
| Rush | 12(15%) | 1(1%) | 6(17%) | 1(1%) |
| Decreased appetite | 11(13%) | 2(2%) | 11(31%) | 3(8%) |
| Urinary tract infection | 8(10%) | 3(4%) | 6(17%) | 3(8%) |
| Reactive capillary hemangiomas | 8(10%) | 1(1%) | 2(6%) | 0 |
| Constipation | 6(7%) | 2(2%) | 11(31%) | 1(3%) |
| Proteinuria | 6(7%) | 0 | 5(14%) | 1(3%) |
| Hypothyroidism | 6(7%) | 0 | 4(11%) | 0 |
| Fatigue | 6(7%) | 2(2%) | 11(31%) | 3(8%) |
| Hyponatremia | 5(6%) | 2(2%) | 3(8%) | 1(3%) |
| Increased blood bilirubin | 5(6%) | 0 | 5(14%) | 1(3%) |
| Increased blood urea | 5(6%) | 1(1%) | 4(11%) | 1(3%) |
| Hypoalbuminemia | 4(5%) | 0 | 4(11%) | 1(3%) |
| Upper respiratory tract infection | 4(5%) | 1(1%) | 4(11%) | 2(6%) |
| Decreased neutrophil count | 4(5%) | 0 | 14(39%) | 11(31%) |
| Decreased white blood cell count | 4(5%) | 0 | 12(33%) | 7(19%) |
| Increased blood alkaline phosphatase | 4(5%) | 1(1%) | 5(14%) | 2(6%) |
| Increased gamma-glutamyl transferase | 4(5%) | 1(1%) | 5(14%) | 1(3%) |
| Thrombocytopenia | 4(5%) | 0 | 7(19%) | 4(11%) |
| Diarrhea | 3(4%) | 1(1%) | 7(19%) | 1(3%) |
| Arthralgia | 3(4%) | 0 | 4(11%) | 1(3%) |
| Hematuria | 3(4%) | 2(2%) | 4(11%) | 1(3%) |
| Nausea | 2(2%) | 0 | 14(39%) | 2(6%) |
| Vomiting | 2(2%) | 0 | 9(25%) | 2(6%) |
| Hyperthyroidism | 2(2%) | 0 | 2(6%) | 0 |
| Asthenia | 2(2%) | 0 | 7(19%) | 1(3%) |
| Any immune related adverse events | 11(13%) | 1(1%) | 8(22%) | 2(6%) |
| Skin adverse reaction | 5(7%) | 1(1%) | 3(8%) | 1(3%) |
| Hypothyroidism | 4(5%) | 0 | 2(6%) | 0 |
| Hyperthyroidism | 2(2%) | 0 | 1(3%) | 0 |
| Colitis | 0 | 0 | 1(3%) | 1(3%) |
| Pneumonitis | 0 | 0 | 1(3%) | 0 |
| Hepatitis | 1(1%) | 0 | 0 | 0 |
| Myocarditis | 1(1%) | 0 | 0 | 0 |
| Myasthenia Gravis | 1(1%) | 0 | 0 | 0 |
| Time to response(week) |  |  |  |  |
| Median(range) | 8.5(4-31) | | 9(1-20) | |

**Supplementary Table S2** The sub-analysis of adverse events and immune-related adverse events in patients treated with PD-1 inhibitor and PD-1 inhibitor plus chemotherapy.
